# The application of CRISPR /Cas mediated gene editing in synthetic biology: Challenges and optimizations

**DOI:** 10.3389/fbioe.2022.890155

**Published:** 2022-08-25

**Authors:** Wenqian Li, Can Huang, Jingyu Chen

**Affiliations:** ^1^ MOE Key Laboratory of Precision Nutrition and Food Quality, College of Food Science and Nutritional Engineering, China Agricultural University, Beijing, China; ^2^ Key Laboratory of Food Bioengineering (China National Light Industry), College of Food Science and Nutritional Engineering, China Agricultural University, Beijing *,* China

**Keywords:** CRISPR/Cas, off-target effects, toxicity, gene-editing efficiency, synthetic biology

## Abstract

Clustered regularly interspaced short palindromic repeats (CRISPR) and its associated enzymes (Cas) is a simple and convenient genome editing tool that has been used in various cell factories and emerging synthetic biology in the recent past. However, several problems, including off-target effects, cytotoxicity, and low efficiency of multi-gene editing, are associated with the CRISPR/Cas system, which have limited its application in new species. In this review, we briefly describe the mechanisms of CRISPR/Cas engineering and propose strategies to optimize the system based on its defects, including, but not limited to, enhancing targeted specificity, reducing toxicity related to Cas protein, and improving multi-point editing efficiency. In addition, some examples of improvements in synthetic biology are also highlighted. Finally, future perspectives of system optimization are discussed, providing a reference for developing safe genome-editing tools for new species.

## Introduction

The features of clustered regularly interspaced short palindromic repeats (CRISPR) were discovered serendipitously in the genomes of various bacteria and archaea during molecular biology studies (Ishino et al., 1987; Mojica et al., 1993). The system was originally used as an adaptive immune defense system by bacteria against the invasion of foreign nucleic acids, such as viruses and plasmids (Ishino et al., 1987; Sovova et al., 2017; Albitar et al., 2018). The immune process is divided into three stages: 1) adaptation: the invading nucleotide fragments are first captured by the host organism, and subsequently, a Cas integrase-derived nucleic acid sequence is inserted into the CRISPR array; 2) biogenesis: the CRISPR array is transcribed into a pre-crRNA (pre-crRNA) containing a spacer and a portion of the repeat, which is subsequently cleaved in the repeat to induce the generation of mature guide crRNA (gRNA); and 3) interference: mature crRNA-Cas complexes recognize specific sites on target nucleic acids through complementary base pairing, triggering Cas enzymes to catalyze the cleavage of effector complexes of invading nucleotides (Debin Zhang et al., 2020; Makarova et al., 2020). CRISPR provides acquired immunity to prokaryotes, in addition to other unrelated functions, such as gene regulation (Mojica and Rodriguez-Valera, 2016). The discovery and application of the CRISPR-Cas enzyme system in prokaryotes and eukaryotes have revolutionized genome engineering by providing directly accessible or editable tools (Barrangou, 2014).

CRISPR/Cas systems usually consist of Cas proteins and CRISPR arrays, in which the Cas proteins are involved in the acquisition and protection of invading nucleotides. Currently, CRISPR/Cas systems are divided into classes 1 and 2 according to the composition of their effectors. Class 1 systems include many Cas protein subunits, including types I, III, and IV (Makarova et al., 2011). In contrast, only one Cas protein involving II, V, and VI subdomains is included in Class 2 (Ishino et al., 2018). Class 2 CRISPR/Cas systems have emerged as an attractive option for developing next-generation genome-editing technologies because of their simple structural design and easy implementation in gene editing and manipulation of cell-free nucleic acids (Tang and Fu, 2018; Debin Zhang et al., 2020). Therefore, we consider a typical Class 2 system as an example to introduce the basic components of the system.

Generally, the CRISPR/Cas9 system is considered the smallest CRISPR/Cas system because its pre-CrRNA processing occurs solely with Cas9, a member of the Cas family. Apart from Cas9, single-guide RNA (sgRNA) is the other main component of this simple and easy Class 2 system. sgRNA structure comprises two parts, one of which plays a role in guiding cleavage by binding to Cas9 (Mojica et al., 2005; Pourcel et al., 2005), whereas the other is capable of binding to the target sequence through complementary base pairing to guide positioning. A conserved sequence called the proto-spacer adjacent motif (PAM) exists downstream of the target sequence (Jinek et al., 2012). The presence or absence of the PAM sequence determines whether Cas9 protein cleaves the target sequence. In this system, Cas9 cleaves the target DNA sequence under the guidance of gRNA to create double-strand breaks (DSB). CRISPR systems in prokaryotes often require the addition of donor DNA fragments because they only have homology-directed repair (HDR) (Liang et al., 1998). In addition, the deletion or insertion of target DNA sequences can also be achieved by the addition of donor DNA fragments. Eukaryotes have access to another mode of non-homologous end joining (NHEJ) repair, allowing bacteria to repair without DSBs in the presence of donor DNA (Lieber, 2010). Another vital element, the Cas9 protein, is regarded as a nuclease with two cleavage domains: RuvC and HNH (Deltcheva et al., 2011). While both domains are activated, the HNH nuclease domain cleaves the DNA strand complementary to the sgRNA, and RuvC cleaves the DNA strand that is not complementary to the sgRNA, forming a nicked double-strand break near the PAM. Based on these properties, Cas9 can be directed to the target gene to generate DSBs by designing the sgRNA spacer sequence. In some studies, the two nuclease domains of RuvC and HNH were mutated to introduce novel Cas9 or completely inactivate Cas9, which has excellent effects in improving target efficiency (Makarova et al., 2011).

**FIGURE 1:**
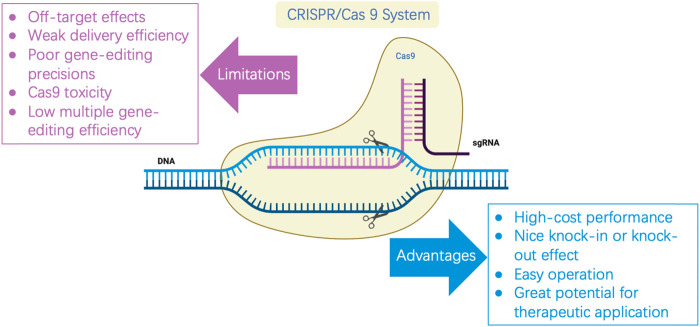
Limitations and advantages of CRISPR/Cas9.

In recent years, the emergence of CRISPR/Cas and its derivative editing technology has become a pillar of experimental biology, as well as a tool for synthetic biology, because of convenient implementation, high mutation efficiency, and great potential for therapeutic applications (Zeballos and Gaj, 2021). However, the system itself has the risk of being off-target, with low multiple editing efficiency. The toxicity of Cas9 affects the broad application of this gene-editing technology in cell factories and synthetic biology (Figure 1). Ensuring the high editing efficiency of the CRISPR/Cas system and reducing off-target effects and toxicity to cells is urgently required to study CRISPR/Cas in new species. In this study, the mechanism of the CRISPR/Cas system and its composition are briefly reviewed. This review focuses on three current problems that have limited the development of this system and summarizes optimization strategies, as well as the latest practical application cases, to provide a reference for more researchers in system optimization and new species development.

## Minimizing off-target editing of CRISPR/Cas system

### Causes of off-target effects

In the CRISPR/Cas system, sgRNA can guide the Cas protein to be cut or modified by recognizing the PAM sequence and binding to the same sequence of the genome. Nucleotide-binding does not require an identical sequence, and the mismatching of a single base pair is acceptable. However, this leads to mismatching between the designed sgRNA and non-target DNA sequence, resulting in unexpected genetic mutation, known as the off-target effect (Haeussler, 2020).

This effect of random cleavage on the genome may considerably limit the development of CRISPR/Cas systems for practical applications. In early studies, Pattanayak et al. found that the specificity of the sgRNA-Cas9 complex depended on the complementary pairing of the 7–12 base sequence of sgRNA adjacent to PAM with the target gene, with distal sequences facilitating mutations, causing unexpected off-target events (Pattanayak et al., 2013). However, many groups using high-throughput sequencing have observed thousands of off-target effects on chromosomes. In some cases, even nucleotide insertions or deletions exist, increasing the off-target range. To address this, the development of genetic sequencing technology and the search for off-target locations *in vitro* using biochemical methods have allowed researchers to determine that most of the off-target sequences are similar to the target sequences, which could be used to forecast possible off-target effects. In prokaryotes, the genome is small with few similar sgRNA sequences, ensuring that the off-target effect has a weak impact. In addition, most prokaryotes only have a homology-directed repair (HDR) repair mechanism, such that DNA double-strand breaks (DSBs) caused by Cas9 are repaired with the assistance of donor DNA. Otherwise, the cells die, further reducing the possibility of off-target effects. Notably, this may also cause mutations in the PAM sequence or its adjacent guide sequence to escape the cleavage by Cas9.

### Strategies to improve on-target effects

In most cases, off-target mutations can be improved by selecting sgRNA with higher specificities. A positive correlation exists between the GC content of sgRNA “seed region” and gene editing efficiency. Moreover, effective cleavage cannot occur with more than three mismatches in sgRNA beyond the “seed region” (Ren et al., 2014). Therefore, when designing sgRNA sequences, sgRNAs with a high GC content and low homology with genomic DNA other than target gene sequences can be selected to improve specificity. The length of sgRNA is also closely related to the targeting efficiency. sgRNA with less than 20 nucleotide sequences can effectively reduce off-target effects without affecting gene editing results. However, the mechanism underlying this strategy is currently unclear (Fu et al., 2014). Various software tools for designing high-precision sgRNAs have been widely developed, mainly focusing on identifying attractive off-target regions in the genome (Martin et al., 2016), allowing for a certain number of mismatches (Haeussler and Concordet, 2016). Many online networking tools, such as CRISPR-2.0 E-Crisp breaking-CAS system (Heigwer et al., 2014; Oliveros et al., 2016; Liu et al., 2017), have been applied to detect off-target mutations, including but not limited to providing services for sgRNA sequence design and reducing the possibility of off-target editing.

In recent years, the modification of wild-type Cas9 has been a crucial strategy to improve CRISPR/Cas9 specificity. This includes the inactivation of a single enzyme digestion domain in Cas9 to obtain NickCas9, which can only cut a single strand, thus requiring the simultaneous participation of two sgRNAs to induce DNA fracture. Therefore, this reduces the off-target rate since the simultaneous misalignment of both sgRNAs can lead to missing the target. Ran et al. utilized this strategy and found that cell lines can significantly reduce the off-target effect by 50–1,500 times and have broad applicability in different cell types (Ran et al., 2013b). Additionally, a series of high-fidelity Cas9 variants, such as xCas9 and SpCas9, can extend the PAM recognition range and DNA specificity, making it another revolutionary tool in gene editing (Zhaohui et al., 2020). Four evoCas9 mutants with improved editing efficiency have also been reported in yeast, showing good targeting and shearing activity. The fidelity of the SpCas9 mutant was found to be 79 times higher than that of the wild-type (Casini et al., 2018). Furthermore, a series of high-fidelity Cas9 variants have also been developed in recent years (Table 1), including Cas9n (Ran et al., 2013a), dCas9 (Qi et al., 2013), spCas9 (Kleinstiver et al., 2016), espCas9 (Cong et al., 2013), Cpf1 (Crosetto et al., 2013), HypaCas9 (Chen et al., 2017), and Sniper-Cas (Lee et al., 2019) (Table 1).

**TABLE 1:** Improved CRISPR/Cas system involving Cas protein, gRNA and donor DNA.

Strategies	Descriptions	Characteristics	Ref
Cas proteins optimization strategy			
xCas9 or Cas9-NG	Engineered versions of *Streptococcus pyogenes* Cas9	Improve target specificity and expand target range	Hu et al. (2018)
Cas9n	Inactivating the HNH or RuvC nuclease domain of Cas9	Edit specific sites	Ran et al. (2013a)
dCas9	Inactivating both HNH and RuvC nuclease domain of Cas9	Base editing without generation of DSBs	Qi et al. (2013)
spCas9-HF1	Mutation of the key amino acid residues of SpCas9 responsible for contact with the target sequence	Variants that reduced nonspecific DNA interactions	Kleinstiver et al. (2016)
espCas9	*Streptococcus pyogenes* K848A, K1003A, R1060A	Requirement of a high specificity	Cong et al. (2013)
Cpf1	Type Ⅱ-Ⅴ CRISPR system	Cpf1 recognizes T-rich PAM, and is degraded by the endogenous protease system after editing	Crosetto et al. (2013)
HypaCas9	Balance nuclease activation and target recognition	Higher genome-wide fidelity	Chen et al. (2017)
Sniper-Cas	An *Escherichia coli* based directed evolution method, Sniper-screen to obtain a Cas9 variant	Higher specificity	Lee et al. (2019)
sgRNA optimization strategy			
Individual promoters/terminators	Each sgRNA has independent promoter and terminator control	High efficiency and wide application, but unstable because of large structure and repeat sequences	Yu et al. (2020)
Type Ⅱ CRISPR crRNA array	Multiple sgRNA expression using crRNA array via one promoter and one terminator	The structure is simple, but the complementary pairing of pre-crRNA and trans-activating crRNA and the intracellular RNase III nuclease need to be considered	Li et al. (2020)
ABEs or CBEs	Transcriptome-wide gRNA-independent editing of RNA bases	Low DNA off-target and indels formation activity	Grunewald et al. (2019)
tRNA processing	Multiple sgRNA are controlled by one promoter and one terminator based on endogenous ribozymes, tRNA processing, and exogenous Csy4 protein	No need to introduce heterologous Cas protein, and showed more stable in multi-site editing	Zhang et al. (2019)
Prime editing (PE)	No DSB or donor DNA is required	89% of known genetic variations associated with human disease can be corrected	Tong et al. (2021)
DONOR DNA optimization strategy			
ssDNA	Single-stranded DNA	Simple to prepare, but limited in length	Lin et al. (2015)
dsDNA	Double-stranded DNA	More stable than ssDNA	Lin et al. (2015)
plasmids	Donor DNA templates are provided as plasmids	The plasmid transformation efficiency is high and stable, but the operation is complicated, and the number of plasmids available in some engineered strains is limited	Zhao et al. (2016)

In addition to optimizing sgRNA and Cas9, reducing the concentration and exposure time of Cas9 are critical strategies to improve specificity. The discovery of anti-CRISPR (ACR) proteins has provided technical possibilities for realizing this strategy. The *ACR* gene expresses the anti-CRISPR-associated protein (ACA), which binds to upstream promoters and regulates *ACR* gene expression during transcription. ACR protein has been shown to inhibit CRISPR/Cas immune function at different stages and prevent Cas9 from binding to DSBs, as well as cleavage, CrRNA loading, or effector complex formation, thus avoiding the continuous expression of Cas9 protein in cells, ultimately reducing miss efficiency (Stanley and Maxwell, 2018). Li et al. demonstrated that the *in vitro* injection of *ACR* genes reduced Cas9-related cytotoxicity and improved transplantation outcomes (Chang Li et al., 2018). Moreover, multiple protein families that naturally inhibited Cas9 have been identified. For example, Pawluk et al. identified three naturally occurring protein families that bind directly to *Neisseria meningitidis* Cas9 (NmeCas9) and act as effective inhibitors of this system in human cells (Pawluk et al., 2016).

Notably, these strategies are mainly based on eukaryotic studies. In contrast, owing to the low off-target rate in prokaryotes, the corresponding optimization strategies have not yet been fully developed in these organisms. However, using these strategies remains an excellent choice as a reference for improving the on-target effects in prokaryotes.

## Minimizing the toxicity of CRISPR/Cas9

**FIGURE 2:**
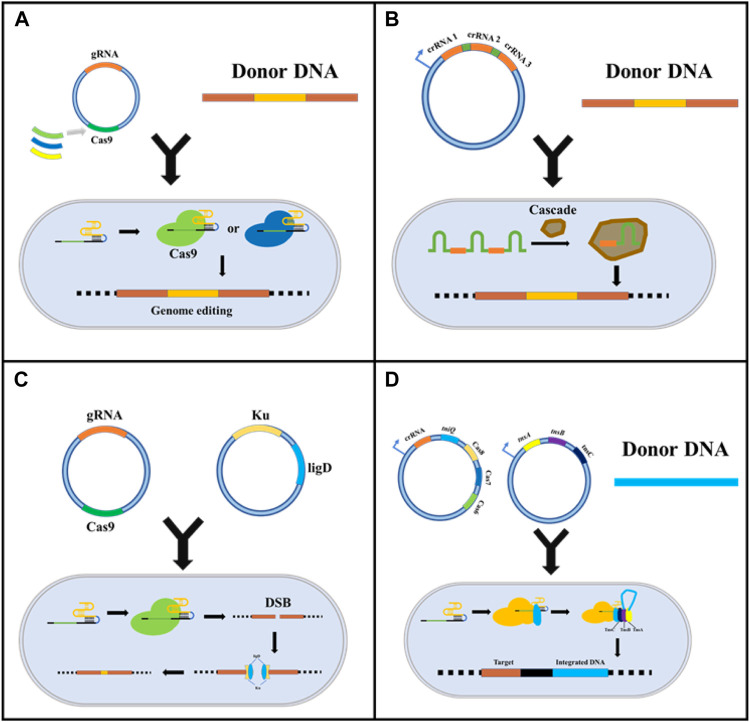
Strategies for reducing CRISPR/Cas9 system cytotoxicity. **(A)** Replacing Cas9 with other less toxic Cas proteins, such as dCas9 and CpfI; **(B)** Using the intracellular endogenous CRISPR/Cas immune system to express crRNA and realized target sites-editing; **(C)** Introducing exogenous NHEJ recombination system to reduce DSB induced cytotoxicity; **(D)** Emerging CRISPR gene editing based on transposons.

The versatility of the CRISPR system has given rise to derivative techniques based on nuclear editing, which may contribute to cytotoxicity owing to the unique nature of prokaryotic gene profiling, especially in microorganisms. CRISPR/Cas can lead to fatal chromosomal breaks, resulting in inefficient transformation and gene editing failure (Jing Zhao et al., 2020). In this review, four methods to mitigate Cas9 toxicity are discussed (Figure 2): substituting for Cas proteins with lower toxicity (McNeely et al., 2010); using endogenous immune systems in prokaryotes to relieve the toxicity, and low transformation efficiency of Cas9 (Luo et al., 2015; Wang et al., 2016; Qin et al., 2021); reducing the cytotoxicity caused by DSBs by introducing the non-homologous end joining (NHEJ) repair mechanism (Huang et al., 2019; Su et al., 2019); and emerging novel CRISPR gene editing based on transposons (Strecker et al., 2019).

Although the CRISPR-Cas9 system from *Streptococcus pyogenes* is widely used in bacterial genome editing, its toxicity has limited research on *Streptomycetes* (Yeo et al., 2019) and *Corynebacterium glutamicum* (Jiang et al., 2017). Thus, establishing CRISPR systems in industrial microorganisms by substituting Cas proteins with an alternative with reduced toxicity is essential. *Cyanobacterium* has been successfully designed and transformed into a strain capable of producing biomass fuels and bioactive substances, such as alkanes and terpenoids (McNeely et al., 2010). However, Cas9 cytotoxicity has hindered the application of the CRISPR/Cas system in cyanobacteria. Unlike Cas9, Cas12a (Cpf1), which is modulated by the lac promoter, can thrive both in crRNA processing and target gene cleavage, resulting in sticky ends (Pattharaprachayakul et al., 2020). Cas12a provides an effective solution for cyanobacteria. However, the reasons for the lower toxicity of Cas12a protein are unknown (McNeely et al., 2010). The same strategy has been applied to *Streptomyces*, in which the FnCas12a protein from *Francisella novicida* was found to be effective (Lei Li et al., 2018). Meanwhile, the author found that the CRISPR-FNCas12A3 system of genetically engineered FnCas12a mutant EP16 can recognize the PAM sequence for precise site mutation and insertion. The CRISPR-FNCAS12A3 system solves the limitations of TTN PAM recognition by *Streptomyces* with a high GC content (Jun Zhang et al., 2020). Additionally, the effector Cas9 (TdCas9_m) from *Treponema denticola*, Cas9 (NmCas9) from *Neisseria meningitides*, and *Corynebacterium glutamicum* codon-optimized Cpf1 (FnCpf1_cg) from *Francisella tularensis* do not affect cell growth (Sun et al., 2018).

In addition to replacing the exogenous Cas protein, using the endogenous CRISPR/Cas system is also an excellent choice to reduce cytotoxicity and the cellular stress and compatibility caused by the external immune system. Luo et al. first attempted to knockout the *Cas3* gene in *E. coli* K-12 by using the endogenous CRISPR/Cas system to enable target gene expression and multiple regulations. Accidental self-targeting is assumed to be forced in the case of *Cas* gene deletion-mediated deactivation of the endogenous CRISPR/Cas system. Specifically, this study used Class 1 CRISPR/Cas systems containing Cascade and Cas3 for DNA targeting. The researchers inactivated Cas3 protein and added a constitutive promoter upstream of the cascade operon, which drove transcription to become individual crRNAs, which could participate in binding DNA and insert into the PAM sequence to inhibit transcription (Figure 1B). This study verified the possibility of endogenous CRISPR system development (Luo et al., 2015). Subsequently, based on this principle, Qin et al. constructed a system for the industrial strain *Gluconobacter oxydans*. They speculated that Cas3 was naturally inactivated by the nucleic acid sequence, and the effectiveness of the system was verified by metabolic engineering. In their study, the endogenous CRISPR interference system (CRISPRi, dCas9 mediated system) was used to study the central carbon metabolic pathway PPP and EDP of *G. oxydans*, achieving flexible and reliable genome editing of *G. oxydans* (Qin et al., 2021).

Unlike eukaryotes, most prokaryotes only have an HDR repair mechanism; thus, DSBs caused by the CRISPR/Cas9 system cannot be repaired in cells that have not successfully transformed donor DNA, giving rise to non-viable cells. Therefore, introducing the NHEJ repair mechanism is vital in reducing DSB-induced toxicity. Huang et al. introduced the NHEJ repair pathway by overexpressing the Ku protein and ligase D derived from *Mycobacterium* in *E. coli*. They constructed an efficient gene knockout system without donor DNA based on CRISPR/Cas9 and demonstrated for the first time the potential of this system in genetic engineering. The researchers reported that the system was able to delete a chromosome fragment of up to 83 kb with an efficiency of over 85%. Notably, this system does not need to rely on efficient, competent cells, possibly because of its lower cytotoxicity compared to other CRISPR/Cas9 systems (Huang et al., 2019). Similarly, Yan et al. innovatively established genome editing tools that reduced cytotoxicity based on CRISPR cutting and NHEJ repair pathways. Ultimately, this resulted in the formation of deletion mutants in *Mycobacterium tuberculosis*, wherein the system had access to double mutations simultaneously, demonstrating its potential for screening a large-scale targeted point in drugs (Yan et al., 2020). In addition to Ku protein and ligase D, Su et al. (2019) found that T4 phage ligase could efficiently repair the NHEJ system alone, enabling *E. coli* to achieve a higher survival rate after producing DSB (Figure 2C).

The transposon is a class of DNA sequences that can be transcribed or reverse transcribed in the genome. The present study has found that a transposase from *Scytonema Hofmanni* can associate with the CRISPR effector (Cas12k) to form CRISPR-associated transposase (CAST). CAST system utilized Cas12k to identify and bind to specific sites in the genome, and directly inserts exogenous gene fragments into target sites by transposase. Cas12k in this system does not revolve in the cleavage of DNA and homologous recombination due to the absence of endonuclease activity. Experimental results showed that the system could successfully insert 2.5 kb DNA fragments at the target site and the success rate is up to 80%, which was far better than the traditional CRISPR system based on HDR (Strecker et al., 2019). Similarly, Klompe et al. also found the Tn7-like transposon in *Vibrio cholerae*. They found that after Cascade recognized the target DNA, Cascade directly combined with the protein (TniQ) to guide the transposon into the target site in the genome. After many experiments, transposons were accurately and completely delivered to the target site of the bacterial genome (Klompe et al., 2019).

## Increasing the target editing efficiency

### Strategies for better gene-editing efficiency

**FIGURE 3:**
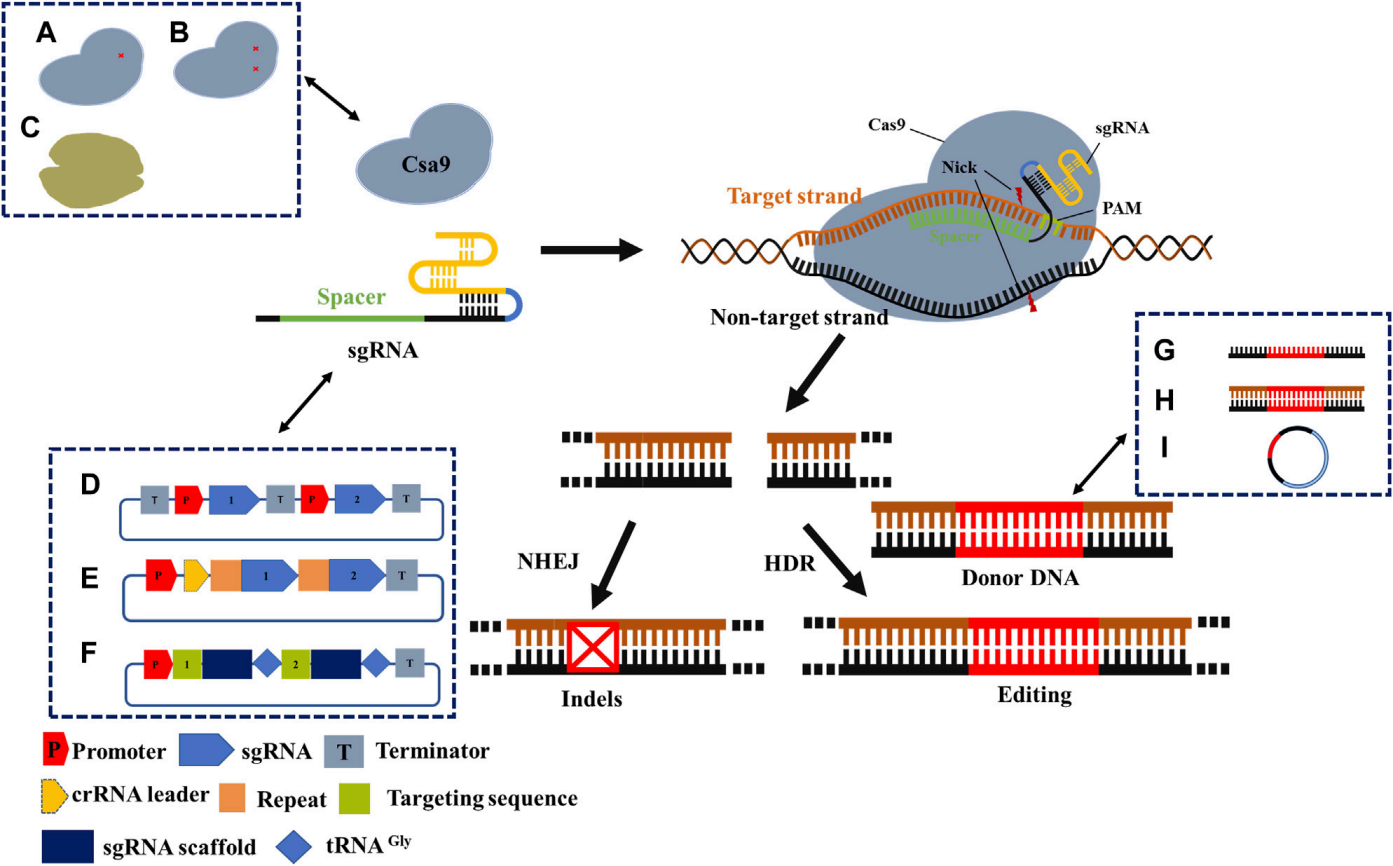
Mechanism and optimization strategy of CRISPR/Cas9 gene-editing system. Cas9 protein cleaves double-stranded DNA under the guidance of sgRNA to obtain DSB. Precise editing by the introduction of donor DNA in cells with HDR machinery, or formation of indels in cells with NHEJ repair machinery. A- C describes general optimization strategies. **(A)** Cas9 nickase; **(B)** Dead Cas9; **(C)** The other Cas proteins. **(D)** Independent expression of sgRNA; **(E)** Simultaneous expression of multiple sgRNAs; **(F)** tRNA process; **(G)** Donor DNA is provided as ssDNA; **(H)** Donor DNA is provided as dsDNA; **(I)** Donor DNA is provided as a plasmid. Recent applications of CRISPR/Cas engineering.

Based on the CRISPR/Cas system components, we distinguished the optimization schemes from Cas protein, gRNA, and donor DNA (Figure 3). Many optimizations may integrate two, all of them, or in combination with other homologous recombination systems, all of which aim to improve the editing efficiency of the CRISPR/Cas system.

Cas9 mutants may be selected to improve the editing efficiency of the CRISPR/Cas system (Table 1). The Cas9 variants mentioned earlier in this review showed a higher specificity than the wild type. Several other examples have been introduced to improve editing efficiency. A variant of the V-type CRISPR system, Cpf1, was promoted, which showed that Cpf1 produced staggered DSBs, followed by the formation of adhesive endpoints. This is more conducive to HDR pathway repair, thus improving the accuracy of gene editing (Zetsche et al., 2015). Additionally, Cpf1 is more sensitive to sgRNA/DNA mismatches, its PAM sequence is more stringent, and Cpf1 is more suitable for targeting T-rich fragments of the genome (Ungerer and Pakrasi, 2016). The CRISPR/Cpf1-RecT system can improve the editing accuracy of PAM sequences, such as TTTC, TTTG, GTTG, or CTTC, reaching an accuracy of over 91.6%. Using the crRNA array, the CPF1-RECT system could simultaneously edit two and three genes, with an editing efficiency of 91.6% for two-gene editing. Using the CPF1-RECT system, the editing efficiency of the 1 kb DNA fragment was 79.6%, while the editing efficiency of 5 kb DNA fragments reached 91.3% (Nannan Zhao et al., 2020). Recently, Wu et al. (2021) discovered a micro-CRISPR-Cas12F system, which solved the problem caused by the large size of the CRISPR/Cas9 complex and promoted the application of viral approaches in therapeutic genome editing (Wu et al., 2021).

Improving the editing efficiency of the CRISPR/Cas system, especially the multiplexed genome engineering efficiency, is beneficial for expanding its application in synthetic biology. Thus, this is a primary focus area for researchers developing novel CRISPR/Cas systems. Multiple editing technologies of CRISPR/Cas have been developed for a wide range of conventional industrial microbes, including *E. coli*, *Streptomyces avermitilis,* and *Bacillus subtilis* (Banno et al., 2018; Zhong et al., 2019; Yu et al., 2020). For instance, by requiring a separate promoter/terminator for each sgRNA transcription unit (Figure 3D), Banno et al. achieved the simultaneous editing of six genes in *E. coli* with an efficiency of 87.5% (Banno et al., 2018). Similarly, Zhong et al. achieved the simultaneous base editing of five (60% efficiency) and nine genes (efficiency not shown) in *Streptomyces avermitilis* (Zhong et al., 2019). Moreover, Yu et al. achieved the simultaneous editing of 3–4 genes in *Bacillus subtilis*, with efficiencies of 100% and 50%, respectively (Yu et al., 2020). In conclusion, this strategy enables high efficiency and wide application in multiplexed base editing, despite shortcomings such as large structure, repeated sequences, and unstable plasmids. Another alternative method can be found in the original CRISPR/Cas system, wherein multiple spacers and repeat sequences can be transcribed through the crRNA array via a promoter and terminator (Figure 3E). Li et al. (2020) applied CRISPR/dCas12a and crRNA arrays in *Corynebacterium glutamate* to inhibit four genes of the lysine synthesis pathway concurrently by CRISPR interference (CRISPRi), whose inhibition exhibited an efficiency of over 90%. Bao et al. applied CRISPR/Cas9 and crRNA arrays to concurrently edit three genes in *Saccharomyces cerevisiae* (Bao et al., 2015). The tRNA-processed type II CRISPR system did not require the introduction of exogenous toxic proteins or shorter sequences (approximately 70 bp) and was more stable in multiplexed base editing (Figure 3F). In another study, the efficiency of simultaneous editing of eight genes was as high as 87% based on multiple sgRNAs used by the tRNA processing system in *Saccharomyces cerevisiae* (Zhang et al., 2019). Specifically, the function of the Cas protein and whether the cognition of sgRNA is mature should be taken into account.

Unlike the application of the sgRNA arrays described earlier, a recent study exported a general, precise genome editing method that detected only a specific sgRNA, denoted as prime editing (PE), using two specific components: the prime guide RNA (pegRNA) and engineered reverse transcriptase. pegRNA can bind to the specific region of DNA that must be edited and obtain a modification template from which it synthesizes DNA with the correct sequence. Notably, DNA repair mechanisms in cells eventually automatically integrate this newly synthesized sequence into their genomes. In contrast to the commonly used CRISPR/Cas system, this system does not require a DSB or donor DNA. In principle, 89% of the known genetic variations associated with human diseases can be corrected by gene editing (Tong et al., 2021).

Moreover, the concentration and delivery method of donor DNA are also critical factors that influence editing efficiency (Table 1). Donor DNA templates can be generated as linear fragments or plasmids. Linear fragments can be divided into linear single-stranded DNA (ssDNA) (Figure 3G) and double-stranded DNA (dsDNA) (Figure 3H). Genome-editing efficiency varies depending on the template provision method. In the case of the *λ*-red recombination system, the system shows a higher editing efficiency when providing templates in the form of ssDNA, which is superior to dsDNA. However, because of the limitation of ssDNA length, long homologous arms cannot be provided; therefore, large fragments cannot be readily knocked out or inserted. In addition to the linear segments, several researchers have chosen the plasmid form to provide donor DNA (Figure 3I). Although the construction of plasmids increases the complexity of the experiments, in prokaryote microbiology, the plasmid conversion efficiency is much higher than that of the linear segment. At the same time, providing the donor DNA plasmid from templates may guarantee the stability of the fragment and extend the period of restructuring effect. Combined with the improvement in the operation process, the editing efficiency and operation time can be significantly enhanced (Lin et al., 2015). When Zhao et al. developed the CRISPR/Cas9 system in *Escherichia coli*, donor DNA was provided in the form of a plasmid (Zhao et al., 2016). After the donor DNA was transferred into the host in the form of a free plasmid, positive transformants were selected for culture, and Cas9 and gRNA expression were induced simultaneously. Cas9 and gRNA were fully expressed in this process, and the donor DNA also acted for a longer time. Ultimately, the process from plasmid construction to obtaining the correct strain was completed within 3 days. Thus, compared to most linear fragment methods in this strain, the process developed by Zhao et al. is more time- and cost-efficient.

Developing chemical-producing cell factories using the CRISPR/Cas system is an important research direction. Compared to traditional gene-editing strategies, CRISPR/Cas technology has been applied to rapidly develop cell factories for various chemicals, owing to its flexibility (Table 2). Owing to the low efficiency of single-point gene editing, many research teams are currently attempting to expand this system. Glucose and xylose are the two most abundant sugars in renewable lignocelluloses. However, they cannot be used simultaneously due to the inhibition of carbon catabolites. Wu et al. developed a set of CRISPRi systems to increase the production of N-acetylglucosamine by repressing xylose utilization genes (zwf, pfkA, and glmM) in *Bacillus* subtilis (Wu et al., 2018). One of the best xylose utilization strains among *E. coli* was obtained using the CRISPR/Cas9-facilitated multiplex pathway optimization (CFPO) technique by Zhu et al. (2017). Four genes (*xyl*A, *xyl*B, *tkt*A, and *tal*B) in the xylose assimilation pathway are regulated by this system. The plasmid, which includes the random RBS library, was first constructed and then co-transformed with pRedCas9, and the original RBS position in the chromosomes was replaced by red-assisted recombination. Next, pgRNA was introduced into the gene plasmid to produce DSBs, eliminating strains that contained native RBS regions. Finally, three transcriptional units were modulated with 70% efficiency (Zhu et al., 2017). Both laid the foundation for further constructing cell factories that efficiently produce chemicals using lignocellulosic resources. In metabolic engineering, a heterologous gene cassette for pyruvate decarboxylase (*pdc*) and alcohol dehydrogenase (*adh*B) from *Zymomonas mobilis* converts pyruvate to ethanol in *E. coli.* Dong et al. (2018) increased ethanol production by three-fold using CRISPR activation (CRISPR A, activation genes), which was optimized to contain a SOXS activator (Dong et al., 2018).

**TABLE 2:** Recent applications of CRISPR/Cas engineering in prokaryotes.

Strains	Stratges	Applications	Efficiency	Ref
*B. subtilis*	CRISPRi and 27 arrays containing sgRNAs with different repression capacities targeting the *zwf, pfkA* and *glmM*	N-acetylglucosamine	The production of N-acetylglucosamine increased 84.1% and a 3-L fed-batch bioreactor reached 103.1 ± 2.11 g/L and 1.17 ± 0.024 g/L/h	Wu et al. (2018)
*E. coli*	CRISPR Cascade system	Alanine	The overall yield in this process was 0.74 g ala- nine/g of glucose, with the yield in the production phase reaching 0.81 g alanine/g of glucose	Ye et al. (2021)
*E. coli*	A multi-gene CRISPRi/a control program	Ethanol	The production quadrupled	Dong et al. (2018)
*E. coli*	CRISPR/Cas9-facilitated multiplex pathway optimization technique (regulating the expression of multiple genes)	Xylose	The utilization rate increased by 3 times	Zhu et al. (2017)
*E. coli*	Multiplex CRISPRi	Malic acid	A 2.3-fold increase	Gao et al. (2018)
*C. glutamicum*	Multiplex CRISPRi	Squalene	3.4 times higher than that of the parental strains	Park et al. (2019)
*C. glutamicum*	Multiplex CRISPRi	L-pyrrolysine	Production improved by 39% compared with wild-type	Park et al. (2018)
*Synechocystis* sp	Multiplex CRISPRi	Octadecanol	Increased octadecanol productivity threefold	Kaczmarzyk et al. (2018)
*K. pneumoniae*	CRISPRi with trp operon	1, 3-propylene glycol and 3-hydroxypropionic acid (3-HP)	Produced 58.9 g/L 3- HP in a 5 L bioreactor	Zhao et al. (2021)

Multiplex CRISPRi via target gRNA arrays and a dCas9 protein have been widely applied to simultaneously repress the expression of multiple genomic DNA loci and has higher efficiency than the CRISPR single gene-editing system. Ye et al. achieved the upregulation of central metabolic enzymes by controlling proteolysis using a combination of the CRISPRi cascade system (Ye et al., 2021). In *E. coli*, Gao et al. used multiplexed CRISPRi to simultaneously inhibit the expression of three crucial enzyme-encoding genes involved in the malic acid biosynthesis pathway. Using this strategy, the titer of the final product, malic acid, was 2.3 times higher than that of the initial strain (Gao et al., 2018). In addition, CRISPRi and sequence-specific sgRNAs successfully achieved single or dual gene inhibition, which increased l-lysine production by 39% compared to that of wild-type *Corynebacterium glutamicum* (Park et al., 2018). Wu et al. have been working on developing synthetic biology using CRISPR as a tool for *C. glutamicum* for several years. Recently, they successfully used CRISPRi to inhibit target genes and achieved a REDOX balance of the precursor, blocking the availability of isoprene diphosphate, resulting in improved *Corynebacterium* that produced squalene from glucose at a rate 3.4-fold higher than the parental strain (Park et al., 2019). Moreover, Kaczmarzyk et al. (2018) used multiplex CRISPRi to allow for the simultaneous partial inhibition of up to six genes, downregulating PlsX (SLR1510) and increasing octadecanol production in *Synechoc*ystis by three-fold (Kaczmarzyk et al., 2018). Beyond these types of strains, several researchers are opting for using CRISPR systems to drive the development of synthetic biology for unfamiliar strains. For example, Zhao et al. (2021) reported that the glycerol oxidation and reduction pathways of *Klebsiella pneumoniae* could be switched to produce 58.9 g/L of 1,3-propylene glycol and 3-hydroxypropionic acid (3HP) by coupling the CRISPRi system with the trp operon (Zhao et al., 2021). Thus, these studies highlight the advantages of the CRISPR/Cas system in gene editing and provide insights into the construction of metabolically engineered strains and the development of cell factories via efficient and accurate modifications of large fragment integration and multi-site editing.

## Exploitation strategy of CRISPR/Cas systems in new species

Currently, the CRISPR/Cas system has been widely developed and applied in commonly studied microorganisms. However, due to some inherent advantages of this system, its development in unconventional hosts is also being carried out based on the three main components (e.g., Cas, gRNA, and donor DNA) required by CRISPR/Cas system. In this context, the system construction strategies can be divided into single-vector expression, multiple-vector expression, and chromosome integration.

Single-vector expression is the most common construction strategy of the CRISPR/Cas gene-editing system, that is, the integration of Cas, gRNA, and donor DNA into the same vector. This strategy has been widely used in the early development of the system combined with GoldnGate molecular cloning techniques, such as CPEC, and has allowed for the shortening of editing cycles. However, its defects lie in the large size of the entire plasmid, which imposes a sizeable physiological burden on cells. Additionally, the plasmid must be imported repeatedly after each round of editing, which requires an effective competent cell. To address this, Huang et al. utilized two promoters (P-thl and P-araE) to express Cas9 and sgRNA in *Clostridium ljungdahlii*, with deletion efficiencies of up to 100% (Huang et al., 2016). Similarly, Xu et al. (2015) developed *Streptococcus pyogenes* CRISPR/Cas9 to edit the genome of *Clostridium cellulolyticum*, which contributed to a high editing efficiency, even when using 200 bp homologous arms.

The multi-vector expression system is used to express CRISPR/Cas components through multiple plasmids, which partly solves the problem of a single-vector expression strategy. Cas can exist in cells for a long time under the control of an inducible promoter. Only gRNA and donor DNA must be constructed for each round of editing, thus addressing the problem of large plasmids in the single-vector strategy, thereby increasing the flexibility of the system. In addition to the two-plasmid system, a strain with many plasmids, such as *E. coli*, can be used to establish a three-plasmid system. Zhu et al. developed a three-plasmid system for Cas9, gRNA, and donor DNA expressed individually, facilitating plasmid construction because of the independent expression vectors. The system also showed high efficiency in multi-site editing because of the continuous expression of the donor DNA (Zhu et al., 2017). Recently, Kozaeva et al. constructed a group of plasmids based on CRISPR and dCas9 in *Pseudomonas putida* that could increase the expression of *glt*A encoding citrate synthase. As a result, *acc*A, an essential gene encoding the subunit of acetyl-CoA carboxylase complex A, was dynamically reduced. The content of acetyl-CoA reconnection was increased by eight times (Kozaeva et al., 2021). Because such multiple plasmids editing systems are limited by the number of plasmids available in the strains, their development in unconventional microorganisms has yet to be fully developed.

The chromosome integrated strategy shows potential for developing strains that do not have multiple available plasmids. However, this strategy involves integrating the Cas gene and gRNA into chromosomes, which requires a traceless operation because of its complex process. In particular, if an iterative cycle system is formed, it is generally necessary to introduce a traceless operating system, leading to a lower prevalence. However, the chromosome integration strategy also has unique advantages, including more stable Cas gene expression, which is less likely to be lost, and the fact that the physiological burden on bacteria is small. In a classic experiment, Westbrook et al. constructed a CRISPR/Cas9 gene-editing box strategy in *Bacillus subtilis* and integrated spCas9 and gRNA expression frames into lacA and thrC sites of chromosomes, respectively, followed by providing dsDNA as a donor DNA template to achieve genome editing. Notably, the researchers achieved high single-point editing efficiency and induced the simultaneous deletion of double genes in the genome, with an efficiency of up to 85% (Westbrook et al., 2016).

## Concluding remarks and future perspectives

In recent years, genome-editing relying on CRISPR/Cas and its derivative technologies, such as precise editing, multi-gene editing, and precise regulation, has experienced rapid innovation, giving rise to comprehensive solutions and greater flexibility in the development of synthetic biology. In turn, this has allowed for large-scale studies of genes and mutations. However, its development has been hampered by issues with editing efficiency due to off-target effects and cytotoxicity, as well as multiple editing efficiency.

The CRISPR/Cas system may be improved via optimization. To this end, a range of techniques have been applied to detect unexpected mutations to improve in-target efficiency and reduce or avoid off-target effects. Additionally, the development of mutants and analogs of Cas9 also represents an effective method to avoid off-target effects, reduce toxicity, and improve editing efficiency. Using ACR competitive binding, the Cas protein can regulate CRISPR expression. Furthermore, selecting ssDNA rather than dsDNA as the donor DNA can ensure the stable existence of fragments and enable a higher editing efficiency. Furthermore, the rise of DNA base editors without DSBs has allowed researchers to solve the problems associated with the original system.

Researchers have continued to focus on improving the CRISPR/Cas system. A recent report found that CRISPR/Cas9 editing produces micronuclei, and chromosomes are structurally deficient, thus initiating a mutation process known as chromothripsis. Chromothripsis is an extensive chromosomal rearrangement limited to one or more chromosomes, causing congenital diseases and cancer in humans. This suggests that chromothripsis is another manifestation of the off-target effects of CRISPR/Cas9 (Leibowitz et al., 2021). Gene-editing technology has been widely applied in microbial synthetic biology and can quickly, efficiently, and accurately create more chassis organisms suitable for the production of high value-added products. CRISPR gene-editing technology may also contribute to the end of the coronavirus pandemic. Synthetic biology and CRISPR/Cas technology are emerging disciplines, albeit with several unresolved issues. However, with the rapid development and in-depth research of these two complementary technologies, improved applications are likely to be obtained in the near future.
